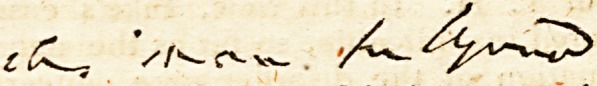# To Correspondents of the London Vaccine Institution

**Published:** 1808-09

**Authors:** John Walker

**Affiliations:** London


### *To the Editors of the Medical and Phyſical Journal.*

Gentlemen,

The notice given on the cover of your Journal, of Vaccine Ichor being supplied, free of expence, by the London Vaccine Institution, continues to keep up unceasing demands for it from all parts of the empire, and from abroad. A London Vulgar has had its confidence much shaken by pamphlets, newspaper paragraphs, and placards stuck about the town; (their authors unworthy the notice of men of science and veracity) but the intelligent part of the public seems every where to have risen above the prejudices produced by these calumniators. Many thousands of charges of matter are distributed by the Society throughout the year; and the very few applicants who may have to complain of not being supplied, have entirely to blame themselves. It is painful on receipt of their urgent applications, not to be able to make out their address. The name is illegibly written, or the place of residence entirely entirely omitted; the means of communication are thus utterly lost, and the small-pox may go on to commit its ravages. To write a bad hand does not betray any want of genius; but, to give a subscription illegibly, shews the greatest want of prudence, if it do not even exhibit a sort of foppery or affectation. I offer you a signature, taken from one of the passports, which, in the last and present century, often opened my way across the guarded barriers of both towns and countries on the continent.

Could Lavater have discovered in it any marks of depth or decision of character? I should rather have supposed it to have been written by an impatient female, (and I draw the observation from real life) than by *Charles Maurice Talleyrand*, that main spring of the revolutionary car of the fickle nation, which in all its motions, whether progressive or retrograde, continues to agitate the whole civilized world, and does not yet show any symptoms of standing still. Your readers, none of them being characters so notorious as *Talleyrand*, let me beg of them in their applications for matter, to write their names so that they may be read by Yours, &c.

JOHN WALKER.

*London*, 15, *viij*, 1808.

## Figures and Tables

**Figure f1:**